# Roastgsa: a comparison of rotation-based scores for gene set enrichment analysis

**DOI:** 10.1186/s12859-023-05510-x

**Published:** 2023-10-30

**Authors:** Adrià Caballé-Mestres, Antoni Berenguer-Llergo, Camille Stephan-Otto Attolini

**Affiliations:** Institute for Research in Biomedicine (IRB Barcelona), The Barcelona Institute of Science and Technology (BIST), Baldiri Reixac, 10, 08028 Barcelona, Spain

**Keywords:** Gene set analysis, Rotation test, Correlation, Competitive test

## Abstract

**Background:**

Gene-wise differential expression is usually the first major step in the statistical analysis of high-throughput data obtained from techniques such as microarrays or RNA-sequencing. The analysis at gene level is often complemented by interrogating the data in a broader biological context that considers as unit of measure groups of genes that may have a common function or biological trait. Among the vast number of publications about gene set analysis (GSA), the rotation test for gene set analysis, also referred to as roast, is a general sample randomization approach that maintains the integrity of the intra-gene set correlation structure in defining the null distribution of the test.

**Results:**

We present *roastgsa*, an R package that contains several enrichment score functions that feed the roast algorithm for hypothesis testing. These implemented methods are evaluated using both simulated and benchmarking data in microarray and RNA-seq datasets. We find that computationally intensive measures based on Kolmogorov-Smirnov (KS) statistics fail to improve the rates of simpler measures of GSA like mean and maxmean scores. We also show the importance of accounting for the gene linear dependence structure of the testing set, which is linked to the loss of effective signature size. Complete graphical representation of the results, including an approximation for the effective signature size, can be obtained as part of the *roastgsa* output.

**Conclusions:**

We encourage the usage of the absmean (non-directional), mean (directional) and maxmean (directional) scores for roast GSA analysis as these are simple measures of enrichment that have presented dominant results in all provided analyses in comparison to the more complex KS measures.

**Supplementary Information:**

The online version contains supplementary material available at 10.1186/s12859-023-05510-x.

## Background

Gene-wise differential expression is the most common analysis of high-throughput expression data generated with microarrays or RNA-sequencing. Subsequent analyses include the screening of the data at broader scales whose measurement unit are groups of genes with common biological functions.

There is a multitude of methods to evaluate aggregated gene expression changes in functional gene sets under different experimental conditions. These are typically classified on the basis of the statistical test being used [1]: (a) self-contained approaches assess whether the observed gene set association with the experimental condition can be expected by chance, without making any reference to other genes in the genome [2–4]; and, (b) competitive approaches aim to determine whether such association with the experimental condition is more extreme than that observed in comparable gene sets in the data [5, 6–12].

Depending on the approach, the distribution underlying the null hypothesis has been approximated non-parametrically based on either gene randomization [6, 13] or sample randomization [2, 3, 6] approaches. Gene randomization is associated with competitive testing whereas sample randomization is presented as self-contained or competitive depending on the test statistic used. Similarly, parametric approximations of either type have previously been developed [4, 8–10].

Gene Set Enrichment Analysis (GSEA) [6], one of the most widely used methods for enrichment in the biomedical community, computes a Kolmogorov-Smirnov-like (KS) test that compares the differential expression effects in genes belonging to the target gene set against the rest of the genes in the genome. For a sufficient number of observations, sample permutations are used to maintain the integrity of the intra-gene set correlation structure in defining the null distribution of the test, resulting in a hybrid approach that combines a competitive statistic with sample randomizations to define the null distribution. However, for small sample sizes (fewer than 7 per experimental condition), p-value granularity becomes a severe problem and gene permutation is recommended instead [14]. This approach, commonly known as GSEAPreranked, overlooks the underlying gene-correlation structure of the testing set, thereby compromising the control of the false positive rate when the intra-gene set correlation exceeds that expected in randomly selected gene sets [15].

Smyth et al. [2] proposed the more general procedure of rotating the residual space of the data, which is useful even for small degrees of freedom. Both the self-contained test (roast) and its competitive version (romer) have been implemented [16]. The romer methodology can be considered the most general gene and sample randomization GSEA approach in the current literature [17], and it is the focus of this work. However, in our opinion, the test statistics provided in romer, which are all functions of the moderated t-statistics ranks, are too limited.

In this paper we review the rotational approach for linear models presented in [18], which motivates the roast method for enrichment, and propose to complete the romer functionality by providing other statistics used in the GSA context. We compare the performance of the KS-based test statistics introduced in GSEA [6] and Gene Set Variation Analysis (GSVA) [19] methodology, as well as re-standardized statistics based on summary statistics measures [7] using both simulated and benchmarking data [20]. Furthermore, as complementary information to interpret the output of the roast GSA methods, we introduce the concept of *effective signature size* as a proxy for the total number of uncorrelated genes in the testing set that can be directly linked to the power of the statistical test being used. All the measures addressed, as well as the approximation of the effective signature size, are implemented in the Bioconductor R package roastgsa.

## Implementation

Source code, documentation and usage example of the S3 R ($\ge$ 4.3.0) package *roastgsa* are available at https://github.com/BBIRBCF/roastgsasource.

### Rotations based gene set enrichment analysis

Rotation tests for multivariate linear regression were first proposed in [18] as generalization of standard permutation tests, with the assumption of multinormality. If such distributional assumption is correct, rotation tests have the great advantage of being applicable to complex models even for small sample sizes. In [2], the rotation approach is adapted to be used as the most general GSEA tool, both for competitive and self-contained testing.

Briefly, the rotation approach consists of the following assumptions and operations: Let $Y_{i}$ be a *q*-dimensional vector, independent for any $i \in [1,...,n]$, that represents the gene expression profile of the *i*th sample with the following multivariate normal distribution assumption:

$$\begin{aligned} Y_i \sim \hbox {MVN}_q(X_iB, \Sigma _r), \end{aligned}$$

where *X* is a $n\times p$ design matrix with $p-k$ adjusting covariates and *k* covariates of interest. The $p\times q$ matrix *B* contains the linear regression coefficients and $\Sigma _r$ is the error covariance matrix. The main steps of the rotational approach proposed in [2] can be summarized by:

1. QR decomposition of *X* to estimate the regression coefficients of interest for the *q* genes and their corresponding error variance.
2. When *q* is sufficiently large, the moderated t-statistic, as defined in the *limma* methodology, can also be computed and used for further calculations of the enrichment score. This t-statistic updates the error variance of the linear models using the information of the estimated variances for all genes based on empirical Bayes posterior means. The prior distribution is obtained by fitting a scaled F-distribution to the sample variances. The posterior distribution is the weighted average of the estimated location of the prior distribution and the sample variances. Weights are determined by the degrees of freedom of the estimated F-distribution and $n-p$, respectively. Moderated t-statistics for all genes are further transformed to z-scores using the quantile function of the Student-t distribution. This is especially useful when the number of degrees of freedom left in the model is small, and the observed t distribution is heavy-tailed.
3. For any testing gene sets, a GSA summary test statistic is calculated using the (z-score transformed) moderated t-values. Depending on the proposed statistic, hypothesis testing is considered either competitive or self-contained.
4. Rotation applied to the residual space of the data can be handled by conditioning only on sufficient statistics of the unknown covariance matrix $\Sigma _{XY}$. Rotation statistics can be estimated and used to define the null hypothesis for hypothesis testing (details in Additional file 3: Sects. 1–2).

The Roast algorithm is implemented in the R package *limma* [16].

### Defining the null hypothesis and GSA summary test statistics

**Fig. 1:**
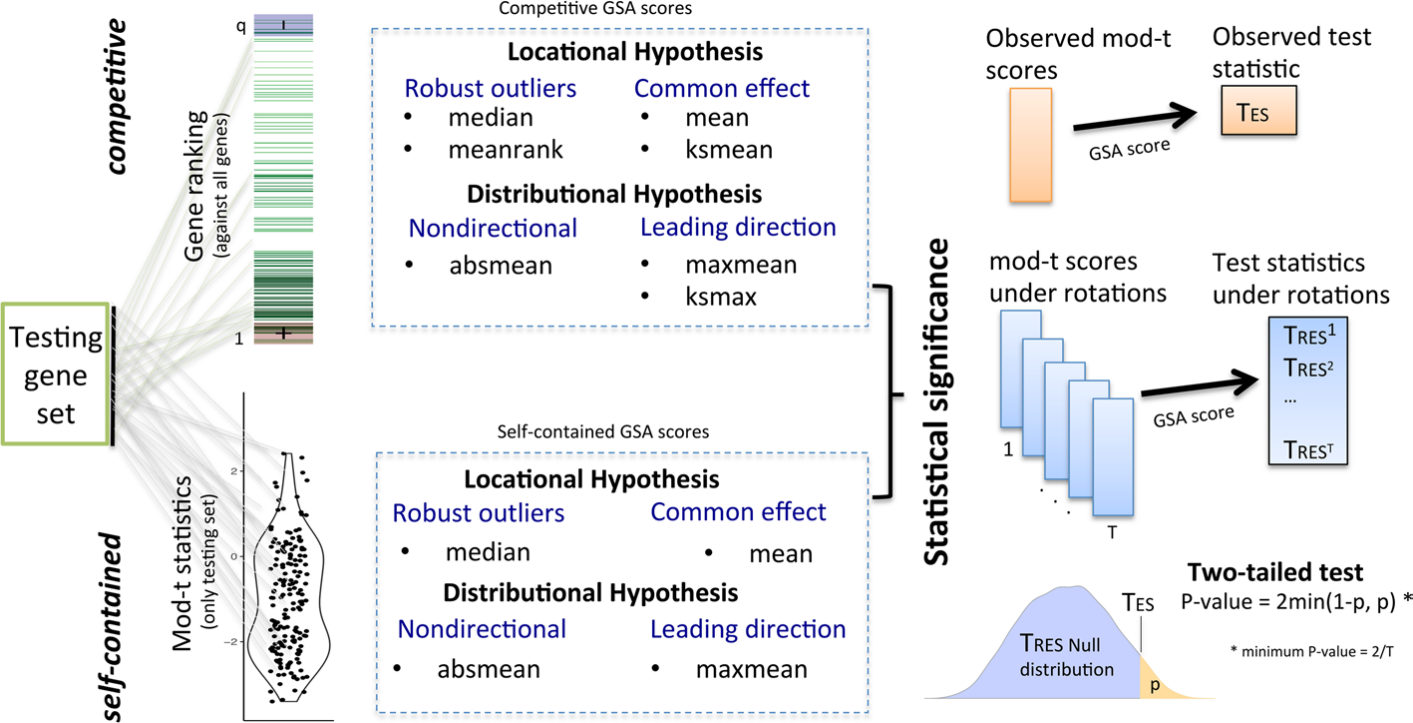
Scope of rotational gene set analysis: from gene set of interest to statistical significance. The enrichment scores mean, maxmean, median and absmean are proposed for both self-contained and competitive approaches. The meanrank, ksmax and ksmean are exclusive scores for competitive testing. All test statistics are defined in Tables 1 and 2

We present several GSA summary statistics with different goals and interpretations that can be used for roastgsa (Tables 1 and 2, Fig. 1). We discriminate between the types of test statistics that can be considered for self-contained hypothesis (SC), in which the observed coefficients of the model for the gene set of interest are compared to what could be found by chance if new data were observed, and competitive hypothesis (CO), in which the evaluation is done after centering and scaling the scores for the gene set of interest against what is observed in the whole genome, thus taking into account the rest of the genome for testing. For both types of hypothesis testing problems, the proposed summary statistics can maintain the integrity of either the distributional or locational null hypothesis or both (Table 3, Fig. 1).

**Table 1:** Formulation of summary statistics mean, absmean, median, and maxmean for both self-contained and competitive testing

	Self-contained score	Competitive score
Mean	$T_{mean}^{SC} = \frac{1}{m_o}\sum \limits _{i\in S}\delta _i$	$T_{mean}^{CO} = \frac{1}{m_o}\sum \limits _{i\in S} \frac{\delta _i - \bar{\delta }}{\hbox {sd}(\delta )}$
Absmean	$T_{absmean}^{SC} = \frac{1}{m_o}\sum \limits _{i\in S} |\delta _i|$	$T_{absmean}^{CO} =\frac{1}{m_o}\sum \limits _{i\in S} \frac{|\delta _i| - \bar{|\delta |}}{\hbox {sd}(|\delta |)}$
Median	$T_{median}^{SC}=\hbox {med}_{i \in S}\delta _i$	$T_{median}^{CO}=\hbox {med}_{i \in S} \frac{\delta _i - \hbox {med}{\delta }}{\hbox {mad}(\delta )}$
Maxmean	$T_{maxmean}^{SC}=\frac{1}{m_o}\sum \limits _{i\in S}\delta _i^*$	$T_{maxmean}^{CO}=\frac{1}{m_o}\sum \limits _{i\in S} \frac{\delta _i^* - \bar{\delta ^*}}{\hbox {sd}(\delta ^*)}$
	$\delta _i^* = \delta _i \hbox {I}[sgn(\delta _i) = sgn(T_{mean}^{SC})]$	$\delta _i^* = \delta _i \hbox {I}[sgn(\delta _i) = sgn(T_{mean}^{CO})]$
	$$[\delta _i] \equiv \hbox {modt-statistics, } i\in \Omega = [1,\ldots ,q]$$	
Notation	$S \equiv \hbox {Testing gene set,} S\subset \Omega , \,\,\, m_0 = |S|,\,\, C = \Omega \setminus S$	
	$$\bar{\delta } = q^{-1}\sum _{i\in \Omega } \delta _i, \,\,\, \bar{|\delta |} = q^{-1}\sum _{i\in \Omega } |\delta _i|, \,\,\, \hbox {med}\delta = \hbox {med}_{i \in \Omega }\delta _i$$	
	$$\hbox {mad} \equiv \hbox {median absolute deviation from the median}$$

**Table 2:** Formulation of enrichment score functions meanrank, ksmax and ksmean, defined for competitive testing

	Competitive score
Meanrank	$T_{meanrank} = \frac{1}{m_o}\sum \limits _{i\in S}\frac{(q+1)/2 - \rho _i}{q}$
Ksmax	$T_{ksmax} = \hbox {I}(A>|a|)A + \hbox {I}(A\le |a|)a,$
	$$A = \max \limits _{l \in S} ks(l|S), a = \min \limits _{l \in S} ks(l|S),$$
	$$ks(l|S) = \frac{\sum \limits _{i\in S} |\gamma _i|^k I(\rho _i \le l)}{\sum \limits _{i\in S} |\gamma _i|^k} - \frac{\sum \limits _{i\not \in S} I(\rho _i \le l)}{q - m_o}$$
Ksmean	$T_{ksmean} = \max \limits _{l \in S} ks(l|S) + \min \limits _{l \in S} ks(l|S),$
	$$ks(l|S) = \frac{\sum \limits _{i\in S} |\eta _i|^k I(\rho _i \le l)}{\sum \limits _{i\in S} |\eta _i|^k} - \frac{\sum \limits _{i\not \in S} I(\rho _i \le l)}{q - m_o}$$
Notation	$[\delta _i] \equiv \hbox {modt-statistics, } i\in \Omega = [1,\ldots ,q]$
	$$[\rho _i] \equiv \hbox {rank for } [\delta _i] \hbox { in decreasing order}$$
	$$S \equiv \hbox {Testing gene set, } S\subset \Omega , \,\,\, m_0 = |S|,\,\, C = \Omega \setminus S$$
	$$\gamma _i = \delta _i - \bar{\delta },\,\,\, \eta _i = (2\rho _i+q+1)/2$$

Specifically, the $T_{mean}$ (both for CO and SC), $T_{meanrank}$ (only CO) and $T_{median}$ (CO and SC) are scores that maintain the integrity of both the distributional and locational hypothesis. The mean is provided to measure the common directional behavior of the testing set. The median and meanrank are robust measures to outliers that can prevent giving importance to gene sets with only a few influential genes at the expense of losing statistical power. These two scores can serve to rank gene sets in battery testing when extreme values are undesirable. We also present the $T_{maxmean}$ (CO and SC), the $T_{ksmax}$ (CO) and the $T_{absmean}$ (CO and SC). These three scores do not control the locational null hypothesis error rates unless the more restrictive distributional hypothesis is imposed. The maxmean uses the moderated t magnitudes of only the most prominent direction, either positive or negative. This is relevant to pick up the main trend of the gene set without compromising statistical power. The ksmax is the original score for GSEA [6], and, similarly to maxmean, it looks for concentration of genes in the testing set in either of the two extremes of the ranked list of genes. The absmean is the only non-directional score presented here which is found useful as a way to capture the activity of highly significative genes in the testing set, regardless of their direction. Finally, the ksmean (CO) uses a similar KS statistic to the ksmax but penalizes effects with contrary directions, hence it controls the rejection rate under the locational null hypothesis when the distributions in the two directions are equal.

**Table 3:** Formulation that distinguishes between distributional and locational hypotheses for both self-contained and competitive schemes

type	Distributional hypothesis	Locational hypothesis
self-contained	$H_{D}: [E[\delta _i] = 0, \forall i\in S]$	$H_{L}: \text {avg}_S(E[\delta _i]) = 0$
competitive	$H_{D}: [F_{\delta _i}, i \in S] = [F_{\delta _i}, i \in C]$	$H_{L}: \text {avg}_S(E[\delta _i]) = \text {avg}_C(E[\delta _i])$
Notation	$[\delta _i] \equiv \hbox {modt-statistics, } i\in \Omega = [1,\ldots ,q]$	
	$$S \equiv \hbox {Testing gene set, } S\subset \Omega , \,\,\, m_0 = |S|,\,\, C = \Omega \setminus S$$	

### Effective signature size of a gene set

Gene sets in publicly available databases, such as in the Broad Hallmarks collection, are specifically built based on modules of coordinated genes [21] (Additional file 1: Fig. S1). Moreover, pathways for other collections such as KEGG or Gene Ontologies might also show high gene-to-gene correlation. This can be attributed to biological co-regulation or to technical biases, which might still be detected even when the effect of the known covariates has been adjusted a priori. Generally, the variance of summary statistics increases with the intra-gene set correlation (Additional file 1: Fig. S2a). This apparent loss of precision implies an incorrect assumption of independence between the genes in a gene set. To capture the degree of this discordance, we define the notion of *effective signature size* of a tested gene set by the total number of genes that are needed, if these were selected at random, to achieve the same summary statistic variance as that of the testing set. The *effective signature size* can be interpreted as a realistic measure of the total number of independent variables that contribute to the variance of the statistic and thus affect the power of the test.

To get an estimate of the effective signature size, sample variances of rotation scores for randomly generated sets of size *m*, $v_{R(m)}^{(l)}$ for any $m \in [1,\ldots ,m_0]$ and any gene set randomization instance $l \in [1,\ldots ,L]$ are compared to the observed rotation scores variance $v_s$ for the tested gene set of size $m_o$ (Additional file 1: Fig. S2b). A* p*-value that approximates the probability of obtaining a variance as extreme as $v_s$ in randomly selected sets of size *m* is computed by:

$$\begin{aligned} \hbox {pval}(m) = 2\min (p_{R(m)},1-p_{R(m)}),\,\, \hbox {with } p_{R(m)} = \frac{1}{L} \sum _{l=1}^L I(v_{R(m)}^{(l)} > v_s). \end{aligned}$$

## Results

**Fig. 2:**
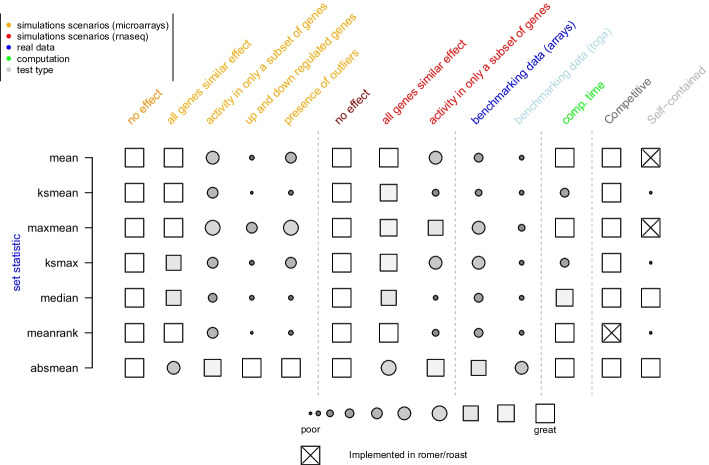
Characteristics of all presented scores: performance in simulated data is measured from 1 (poor) to 10 (great) based on the obtained recovery rates (the average recovery rate relative to the best rate); performance in benchmarking data is measured from 1 to 10 based on the M1 ranking; computational time is measured relative to the fastest method; Scores that were implemented in limma are specified for both romer (competitive scores) and roast (self-contained scores) functions

Specification and performance comparisons of the presented *roastgsa* statistics is provided in Fig. 2. Full results and discussion are detailed below.

### Comparison of statistics in simulated data

#### Microarrays simulation model

We simulate data following a multivariate normal distribution, i.e.,

$$\begin{aligned} y_i \sim N_p(\beta X_i, \Sigma ), { \hspace{0.3cm}} i \in [1,\ldots ,n], \end{aligned}$$

with $X_i = 0$ for $i \le n/2$ and $X_i = 1$ for $i > n/2$. With the objective to use a gene-to-gene linear dependence structure that could be observed in a real case study, the covariance $\Sigma$ is determined by shrinking the sample correlation matrix of the metabric data (Additional file 3: Sect. 3) by the Identity matrix (to find a positive definite matrix). The expected values $\beta X_i$ for all genes measured in the metabric data are specified with regards to the scenario of testing under consideration (simulation scenarios are presented below).

#### RNA-seq simulation model

We consider the gene expression counts matrix observed in the GTEX-Breast samples, from mammary tissue (Additional file 3: Sect. 3), and randomly assign *n* samples to two groups of size *n*/2 and add signal to the initial counts using the binomial thinning approach implemented in the *seqgendiff* R package [22], function thin_2group. Log2-fold changes for all genes measured in the GTEX-Breast data are specified with regards to the scenario of testing under consideration (simulation scenarios are presented below). Matrix counts are log-transformed with regularization using DESeq2 [23], rlog function, prior to *roastgsa* testing.

#### Evaluation criteria

We take 1000 instances of the simulation process with $n = 6, 10, 20, 30, 100$ (sampling multivariate normal data for microarrays or downsampling GTEX-Breast counts + binomial thinning for RNA-seq). From these *n* samples, the condition of interest is determined by a factor variable that takes values 0 and 1 randomly (*n*/2 times each).

Moderated t-statistics are estimated for each instance of the simulation process. We use 500 rotations for approximating the *p*-values. To evaluate the performance of the *roastgsa* scores, we compute the proportion of times (from the total 1000 instances) that the test is rejected at a significance level of 0.05.

#### Simulation scenarios

We consider five different biologically meaningful scenarios to evaluate the performance of the methods (Additional file 1: Fig. S3):

- (SC0) There is no effect of the condition of interest on the expression of the tested gene set.
- (SC1) All genes in the tested gene set have the same expected fold change, which is larger than the global expected fold change.
- (SC2) Only a group of interconnected genes in the gene set have a common activity in the gene set.
- (SC3) Two groups of genes, one up-regulated and the other down-regulated, are active in the gene set.
- (SC4) Few genes present a much higher effect than the rest of the genes (outliers).

SC0 is a clear consideration of a model under the null hypothesis to evaluate the empirical size of the test. SC1 and SC2 could be strategies to evaluate the power of the test, as target gene sets under these two models are likely to be considered biologically relevant. SC3 occurs less frequently in public databases but its recovery might also be useful to researchers. Targeting gene sets under SC4 is slightly more undesirable.

To mimic biologically realistic correlation structures of the test gene sets in microarrays simulations, we consider two gene sets from the literature that show substantially different intra-gene set correlations (Additional file 3: Sect. 3): genes in the (A1) TNFA signaling via NFKB hallmark with a mean correlation of 0.10, and genes in the (A2) interferon alpha hallmark with a mean correlation of 0.27 (Additional file 1: Fig. S1). Besides these two pathways, we consider an artificial case control with 31 uncorrelated genes (A3). For RNA-seq data, we consider only the SC0, SC1 and SC2 scenarios. For SC1 and SC2, we take two distinct gene sets, one with a cluster of highly correlated genes (average correlation of 0.22) and another with randomly selected genes (with an average correlation near 0). Importantly, for the simulations, we assume that all genes remain either unchanged or are affected equally by the condition, with the exception of the genes in each test gene set considered, which are enriched (as specified in Additional file 2: Table S1–S2), one gene set at a time in independent simulation instances.

#### Performance of statistics using simulated data

Recovery rates for SC0-SC4 are compared across *roastgsa* scores, and the complete tables are presented in Additional file 2: Tables S3–S9. False positive rates are controlled for all presented scores. In terms of statistical power, on the one hand, scores that aim to capture the common activity of the pathway, such as the mean, ksmean or mean rank, do well in SC1 but fail to find good recovery rates for scenarios such as SC2, SC3 and SC4, where only a few genes from the whole testing set are differentially expressed. On the other hand, the maxmean and absmean do not penalize for non-global activity, as it happens in more democratic scores such as the mean or meanrank, leading to the largest recovery rates for SC2, SC3 and SC4. Strikingly, the absmean score loses power with respect to the maxmean approach for structures with low correlation (A3 in microarrays and SC2-lowcor in RNA-seq). Finally, the ksmax provides poorer recovery rates than the maxmean, with the latter defining a much simpler statistic for interpreting the outcome. These results are confirmed in both RNA-seq and microarrays data.

### Comparison of competitive statistics in real data

#### Microarray and RNA-seq benchmarking data

The *GSEABenchmarkeR* package [24] facilitates 42 datasets that are part of the GEO2KEGG microarrays compendium [20], in which investigated phenotypes were associated with specific diseases. Additionally, the *GSEABenchmarkeR* provides 16 TCGA datasets with the gene expression (RNA-seq) profile for patients with different types of cancer and also for a few samples with adjacent normal tissues. For each dataset, the relationship of several KEGG gene sets with the disease under investigation was rated by a “relevance score”(MalaCards, [25]), with the highest scores corresponding to gene sets largely associated with the disease.

These data have served as a benchmark to compare the performance of the presented GSA test statistics for battery testing. The outcomes of the *roastgsa* are ranked from the most significant ($I = 1$) to the least significant ($I = p$) gene set and are compared to the MalaCards relevance scores (which we denote by $\rho$) using the following measures:

$$\begin{aligned} M1 = \sum _{i=1}^p \rho _i \,(1-I(i)/p);\,\, M2 = \sum _{i\in T} \rho _i \,(1-I(i)/p), \,\, i \in T \hbox { if } I(i) <= 50. \end{aligned}$$

The measure M1 uses the ranks of all pathways in a weighted average whereas in M2 only the top 50 pathways contribute to the performance measurement. This second measure is proposed to reduce the importance of gene sets at the bottom of the rankings, which tend to be overlooked when doing screenings of battery testing.

To evaluate the performance of the roastgsa approach, we first compare the M1 measure to what could be obtained if gene set rankings were found by chance. This was done by permuting the order of the gene set outcomes 1000 times. A p-value was calculated as the percentage of cases with the observed value being inferior to the permutation-based instances.

For the microarrays compendium, since relevance scores from different datasets are difficult to compare [24], for every dataset, we rank the performance of the seven GSA test statistics (from best 1 to worst 7) based on their M1 (and M2) ratings.

#### Performance of statistics using benchmarking data

In microarray data, the absmean score achieves the most similar rankings to the benchmarking data of all the studied methods, with the maxmean being slightly better than the ksmax (Additional file 1: Fig. S4–S7, Additional file 2: Table S10).

In the RNA-seq data, the absmean is the only score that obtains satisfactory rankings (9 out of 16 datasets have more extreme values of M1 than expected by random permutations, with $\alpha = 0.10$). The rankings for the rest of the scores are poor, with only 2 out of the 16 datasets presenting M1 measures not expected at random (Additional file 1: Fig. S8).

### Computational complexity

In terms of computational complexity (Table 4), the absmean, mean, maxmean, and meanrank are the fastest scores to compute. The median statistic requires slightly more time than the mean while the KS-based statistics take considerably longer times than the other summary statistics.

**Table 4:** Computational time (system.time R function outcome) for all proposed scores

	Elapsed	Relative	User.self	Sys.self
Absmean	12.814	1.000	12.567	0.183
Mean	12.865	1.004	12.612	0.210
Maxmean	14.131	1.103	13.825	0.249
Meanrank	16.585	1.294	15.714	0.811
Median	33.613	2.623	33.046	0.488
Ksmax	64.745	5.053	63.332	1.337
Ksmean	114.006	8.897	111.758	2.178

Execution time obtained by using 10 replications of roastgsa on 50 gene sets, 500 rotations and $N = 50$ (25 per group). Ksmax and ksmean are computationally much more intensive than the other summary statistics

### Visualization of roastgsa results

**Fig. 3:**
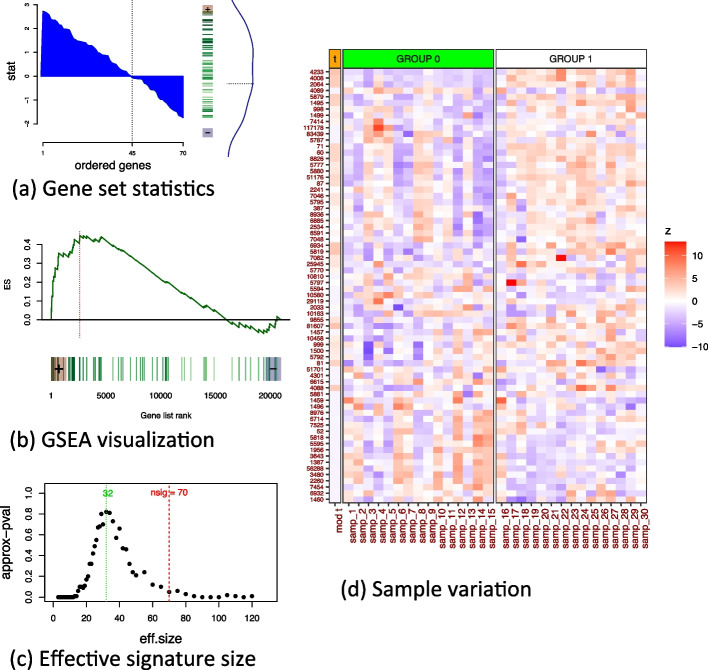
*Roastgsa* output figures: **a** the ordered moderated t-statistics in various formats: area under the curve for all genes ordered by moderated-t statistic, barcode plot for these ordered values and density; **b** classic GSEA plot **c** effective signature size *p*-value curve that determines the number of randomly selected genes needed to obtain levels of variability in the rotation GSA scores as extreme as the rotation GSA scores variance in the testing gene set; **d** normalized expression values and gene set statistics to represent the variation across samples for the gene set of interest

In the *roastgsa* R package, we implement several alternatives to visualize the results. The moderated t-statistics observed in the gene set of interest, which are centered and scaled when considering competitive testing, are represented as in Fig. 3a. This representation can be easily linked to the GSA test statistics used for enrichment. For example, the mean score can be related to the difference between the area for the positive scores and the area for the negative ones (separated by the dashed vertical line), or the maxmean can be characterized by the largest area (either the area with positive scores or the area with negative scores). The KS random walk enrichment plot associated with classic GSEA is still the most frequent representation for this type of enrichment analysis. Although this representation can be difficult to relate to simple summary statistics, we also included it as part of the *roastgsa* outcome (Fig. 3b). A *p*-value curve with the effective signature size (Fig. 3c) is helpful for linking the statistical significance of the tested set with the trend observed for all moderated t-statistics shown in Fig. 3b–c. For instance, a strong tendency in either side of a large part of the genes in the set of interest might not always correspond to statistically relevant results when genes are strongly correlated. This is due to the variance of the test statistics under rotations decreasing with the effective signature size, not the signature size itself. This graphical visualization is provided to guide the interpretation of the results. We complete the *roastgsa* outcome with a heatmap that shows a full landscape of the gene set activity of the testing set (Fig. 3d).

## Conclusions

This work reviews the rotation testing approach for gene set analysis and compares the performance of the method under different enrichment score measures using both simulated and benchmarking data. The absmean (non-directional) and maxmean (directional) scores are simple measures of enrichment that present dominant results in all provided analyses in comparison to the more complex ksmax measure. Similarly, the mean or meanrank statistics find similar powers to ksmean, the latter being much more computationally challenging. Following these empirical results, and also given the conclusive results in the work by [7], we encourage the use of simpler measures for GSA such as the (leading method in our comparison) absmean, or the directional scores maxmean and mean.

Choosing between the absmean, maxmean, or mean should depend on the type of gene sets that are given priority for recovery. We distinguish between these two clear scenarios: [A] common activity in all genes; and [B] a few active genes but with large effect sizes. In our simulations, we presented one case under A (SC1) and three distinct cases under B (SC2-4). The absmean score would favor gene sets under scenario B over gene sets under A. In fact, we observed that the absmean score could lose power with respect to the mean score due to a combination of both low effect size and a relatively high percentage of activated genes (Additional file 3: Sect. 4). On the other hand, there are the mean, meanrank and ksmean scores, which are designed to maximize recovery under the scenario A but have limited capacity to detect hits under scenario B. The maxmean falls in the middle and tends to be the second best in the two types of scenarios in our simulations. In the benchmarking data, some KEGG pathways contain both activator and inhibitor genes, which might explain why the absmean score outperforms the other scores evaluated.

We considered both microarrays (assuming multinormality) and RNA-seq data in simulation scenarios resembling real case studies. One aspect that we explored further in the RNA-seq data was the relationship between gene coverage and power for the roastgsa methods. For a fixed effect size, the power to detect differentially expressed genes increases with the total coverage. Consequently, true enriched gene sets with a higher percentage of lowly expressed genes are less likely to be detected at the same significance level as a gene set of the same size and higher overall expression (Additional file 3: Sect. 5).

Although the main focus of this work was the comparison of the *roastgsa* scores using the roast rotations algorithm to define the null distribution, we also examined the performance of a widely used GSA approach, namely the *limma* method *camera*. We compared the *roastgsa* and *camera* competitive approaches using the benchmarking data. We found that the absmean and the maxmean approaches (*roastgsa*) outperformed the *camera* method, which found comparable results to the *roastgsa* mean approach (Additional file 3: Sect. 6).

Commonly used GSEA plots typically provide information regarding gene variation after averaging out the sample variability (i.e., taking gene-wise fold changes or t-statistics as shown in Fig. 3a–b). We highly recommend complementing these plots with a graphic that also allows visualization of sample variability for the tested gene sets. If the dimensions are not too large, a simple heatmap, as shown in Fig. 3d (result from *roastgsa* R package), is useful to detect those genes that are activated in the process, as quality control to detect samples that can be highly influential in the analysis, and last and foremost, as a way to be honest with the total amount of data that is available for testing.

### Supplementary information

**Additional file 1: **supplementary figures.

**Additional file 2:** supplementary table.

**Additional file 3:** some supplementary material: comprehensive description of rotation approach for geneset enrichment analysis; description of public data considered; comparison between absmean and mean statistics in simulated data; importance of gene coverage for roastgsa methods; and comparison between camera and roastgsa approaches in benchmarking data.

## Data Availability

The datasets used and/or analyzed during the current study are available publicly. Analyses can be reproduced following source code in https://github.com/BBIRBCF/roastgsasource, covariance matrix used for simulations is available from the corresponding author on reasonable request.

## References

[1] Goeman JJ, Buhlmann P (2007). Analyzing gene expression data in terms of gene sets: methodological issues. Bioinformatics.

[2] Lim E, Wu D, Smyth GK, Asselin-Labat M-L, Vaillant F, Visvader JE (2010). ROAST: rotation gene set tests for complex microarray experiments. Bioinformatics.

[3] Nam D (2011). De-correlating expression in gene-set analysis. Bioinformatics.

[4] Larson JL, Owen AB (2015). Moment based gene set tests. BMC Bioinf.

[5] Barry WT, Nobel AB, Wright FA (2005). Significance analysis of functional categories in gene expression studies: A structured permutation approach. Bioinformatics.

[6] Subramanian A, Tamayo P, Mootha VK, Mukherjee S, Ebert BL, Gillette MA, Paulovich A, Pomeroy SL, Golub TR, Lander ES, Mesirov JP (2005). Gene set enrichment analysis: a knowledge-based approach for interpreting genome-wide expression profiles. Proceed Natl Academy Sci.

[7] Efron B, Tibshirani R (2007). On testing the significance of sets of genes. Annals Appl Statist.

[8] Wu D, Smyth GK (2012). Camera: a competitive gene set test accounting for inter-gene correlation. Nucleic Acids Res.

[9] Kim SY, Volsky DJ (2005). PAGE: parametric analysis of gene set enrichment. BMC Bioinf.

[10] Luo W, Friedman MS, Shedden K, Hankenson KD, Woolf PJ (2009). GAGE: generally applicable gene set enrichment for pathway analysis. BMC Bioinf.

[11] Yaari G, Bolen CR, Thakar J, Kleinstein SH (2013). Quantitative set analysis for gene expression: a method to quantify gene set differential expression including gene-gene correlations. Nucleic Acids Res.

[12] Mishra P, Törönen P, Leino Y, Holm L (2014). Gene set analysis: limitations in popular existing methods and proposed improvements. Bioinformatics.

[13] Sergushichev A (2016). An algorithm for fast preranked gene set enrichment analysis using cumulative statistic calculation. bioRxiv.

[14] GSEA-MSigDB Documentation. https://docs.gsea-msigdb.org/. Accessed: 2023-01-30

[15] Tamayo P, Steinhardt G, Liberzon A, Mesirov JP (2016). The limitations of simple gene set enrichment analysis assuming gene independence. Stat Methods Med Res.

[16] Ritchie ME, Phipson B, Wu D, Hu Y, Law CW, Shi W, Smyth GK (2015). limma powers differential expression analyses for RNA-sequencing and microarray studies. Nucleic Acids Res.

[17] Rahmatallah Y, Emmert-Streib F, Glazko G (2016). Gene set analysis approaches for RNA-seq data: performance evaluation and application guideline. Briefings Bioinf.

[18] Langsrud Ø (2005). Rotation tests. Stat Comput.

[19] Hanzelmann S, Castelo R, Guinney J. GSVA: gene set variation analysis for microarray and RNA-Seq data **14**(1), 7 (2013). 10.1186/1471-2105-14-7 10.1186/1471-2105-14-7 PMC3618321 23323831

[20] Tarca AL, Bhatti G, Romero R (2013). A comparison of gene set analysis methods in terms of sensitivity, prioritization and specificity. PLoS ONE.

[21] Liberzon A, Birger C, Thorvaldsdóttir H, Ghandi M, Mesirov JP, Tamayo P (2015). The molecular signatures database Hallmark gene set collection. Cell Syst.

[22] Gerard D (2020). Data-based RNA-seq simulations by binomial thinning. BMC Bioinf.

[23] Love MI, Huber W, Anders S (2014). Moderated estimation of fold change and dispersion for RNA-seq data with DESeq2. Genome Biol.

[24] Geistlinger L, Csaba G, Santarelli M, Schiffer L, Ramos M, Zimmer R, Waldron L. GSEABenchmarkeR: Reproducible GSEA Benchmarking. (2019). R package version 1.2.1. https://github.com/waldronlab/GSEABenchmarkeR

[25] Rappaport N, Twik M, Plaschkes I, Nudel R, Stein TI, Levitt J, Gershoni M, Morrey CP, Safran M, Lancet D (2017). MalaCards: an amalgamated human disease compendium with diverse clinical and genetic annotation and structured search. Nucleic Acids Res.

