# Supplementary material for: Roastgsa: a comparison of rotation-based scores for gene set enrichment analysis
Source: BMC Bioinformatics. 2023 Oct 30;24:408. doi: 10.1186/s12859-023-05510-x (PMC10617084; doi:10.1186/s12859-023-05510-x)
Supplement: Supplementary file 1 — Additional file 1: supplementary figures. [file 12859_2023_5510_MOESM1_ESM.pdf]

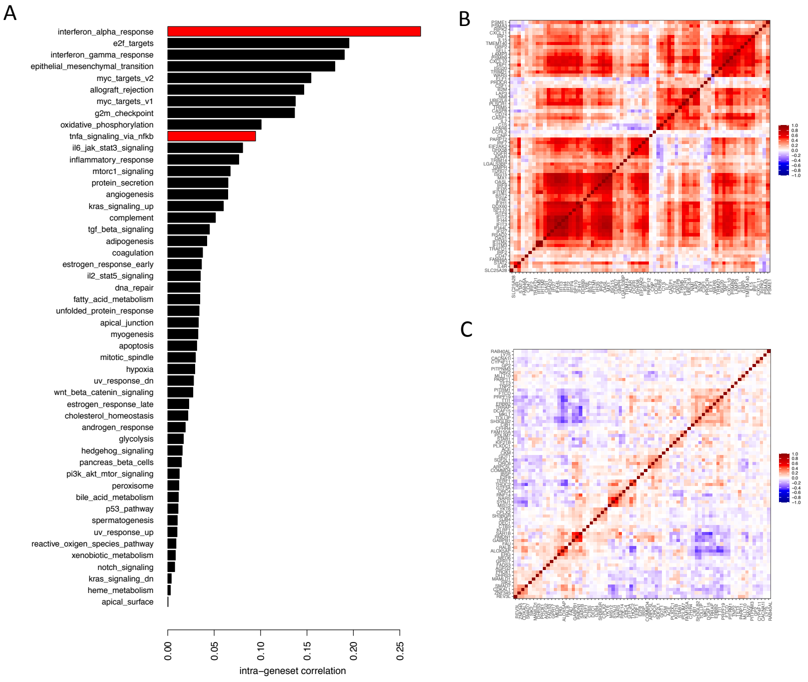

Figure S1: [A] Intra-gene set correlation levels for all Hallmarks gene sets measured by the average correlation across genes of the testing set. Interferon alpha response, with the highest average correlation, and TNFA signaling via NFKB, with an intermediate correlation, are used to define correlation structures in simulated studies. [B] Heatmap with the interferon alpha response gene-gene Pearson correlations (from -1 in dark blue to 1 in dark red). [C] Heatmap with a random signature (of the same size as Interferon alpha response) gene-gene Pearson correlations (from -1 in dark blue to 1 in dark red) showing much milder correlations than the structure observed in B.

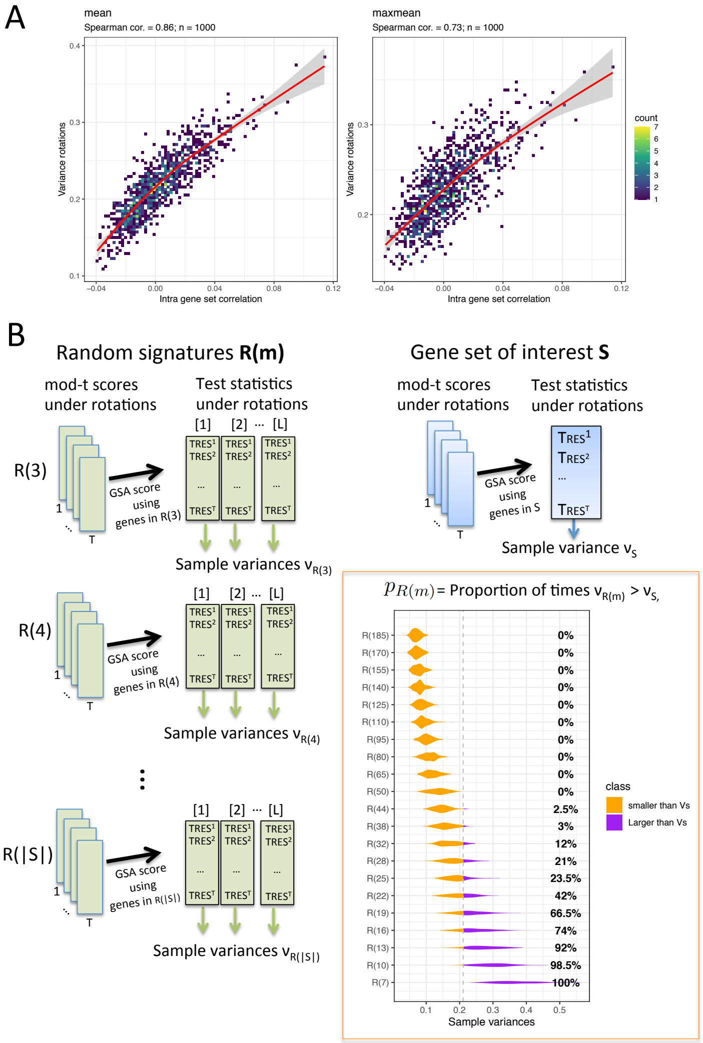

Figure S2: [A] Relationship between the intra gene set correlation and the estimated variance for scores (mean or maxmean) under rotations using 1,000 randomly generated signatures of size 20; The observed loss of precision in the most highly correlated sets can be linked to a decrease in the effective signature size; [B] The estimation of the effective signature size is based on comparing the null distribution (which we characterized by the variance of rotations scores) generated in the testing set S against null distributions that could be generated under random signatures of several sizes. A measure of evidence can be calculated using the proportion of times the variances for the random sets exceed the variance for the testing set.

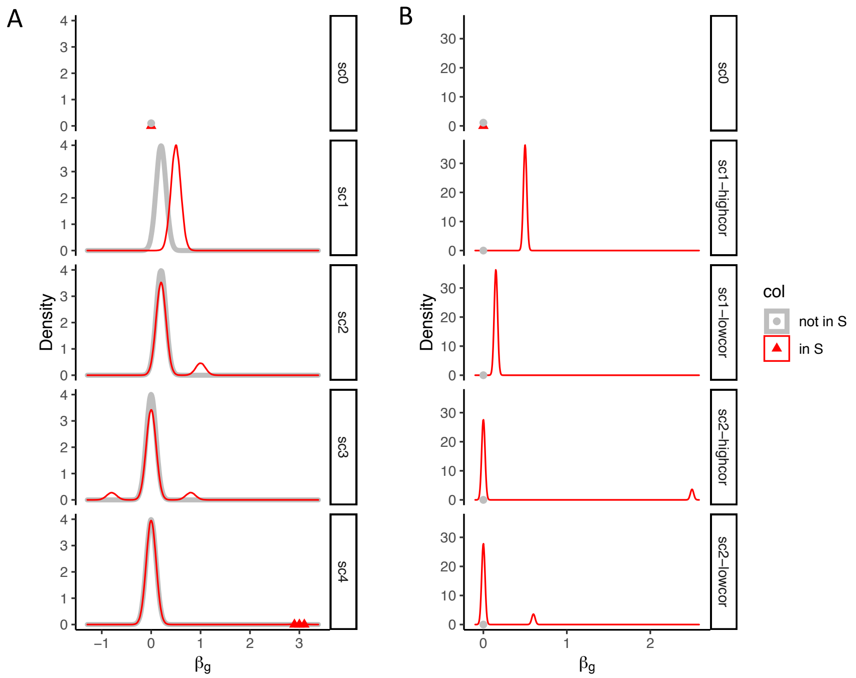

Figure S3: Simulated scenarios for [A] microarrays type of data and [B] RNA-seq type of data. The coefficient β determines the log2 fold change, for all genes, colors distinguish among genes in and not in the testing gene-set S. SC0 is the scenario under the null hypothesis where all genes have exactly the same fold change. SC1-SC4 are scenarios to evaluate the power of the roastgsa statistics. Exact parameter specifications are provided in Additional file 2:Table 1-2.

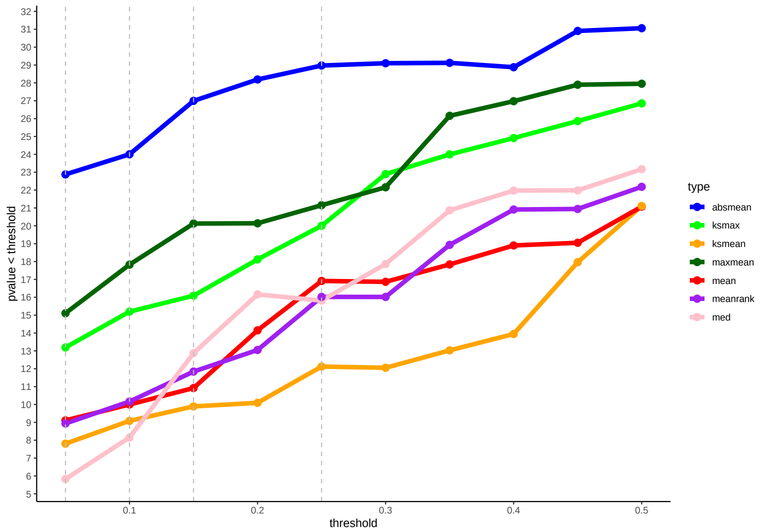

Figure S4: Number of datasets (out of the 42 GEO2KEGG microarray benchmarking datasets) with a M1 observed measure higher than expected by chance. In the x-axis there is the percentage of randomly generated rankings that present the same M1 score or higher than the observed score. The absmean finds the best results, followed by the maxmean.

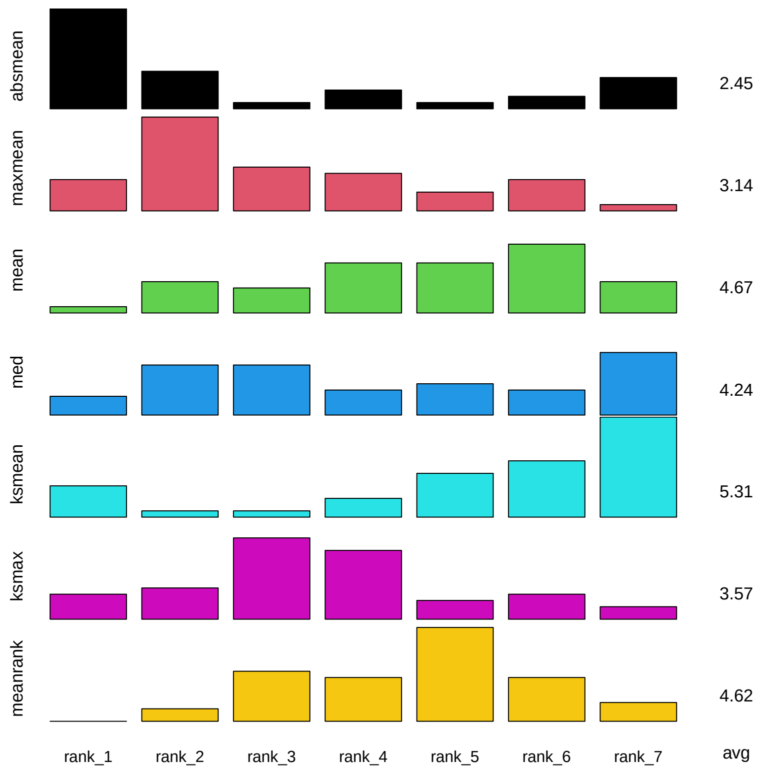

Figure S5: Barplot and average ranks resulting from measure M1 using the 42 datasets from the GEO2KEGG microarray compendium. Rank 1 is the highest rate and rank 7 is the lowest rate. Only competitive scores are used. The absmean scores achieve the best rates, followed by the maxmean and the ksmax. The meanrank and ksmean scores find the worst rates.

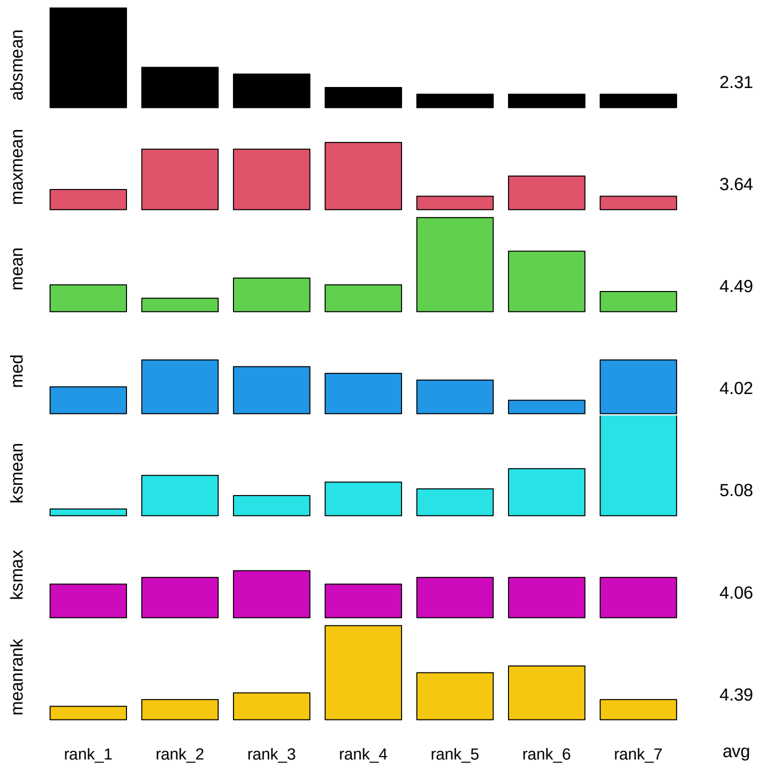

Figure S6: Average rank resulting from measures M2 using the 42 datasets from the GEO2KEGG microarray compendium. Rank 1 is the highest rate and rank 7 is the lowest rate. The absmean score achieves the best rates in the two measures. Only competitive scores are used.

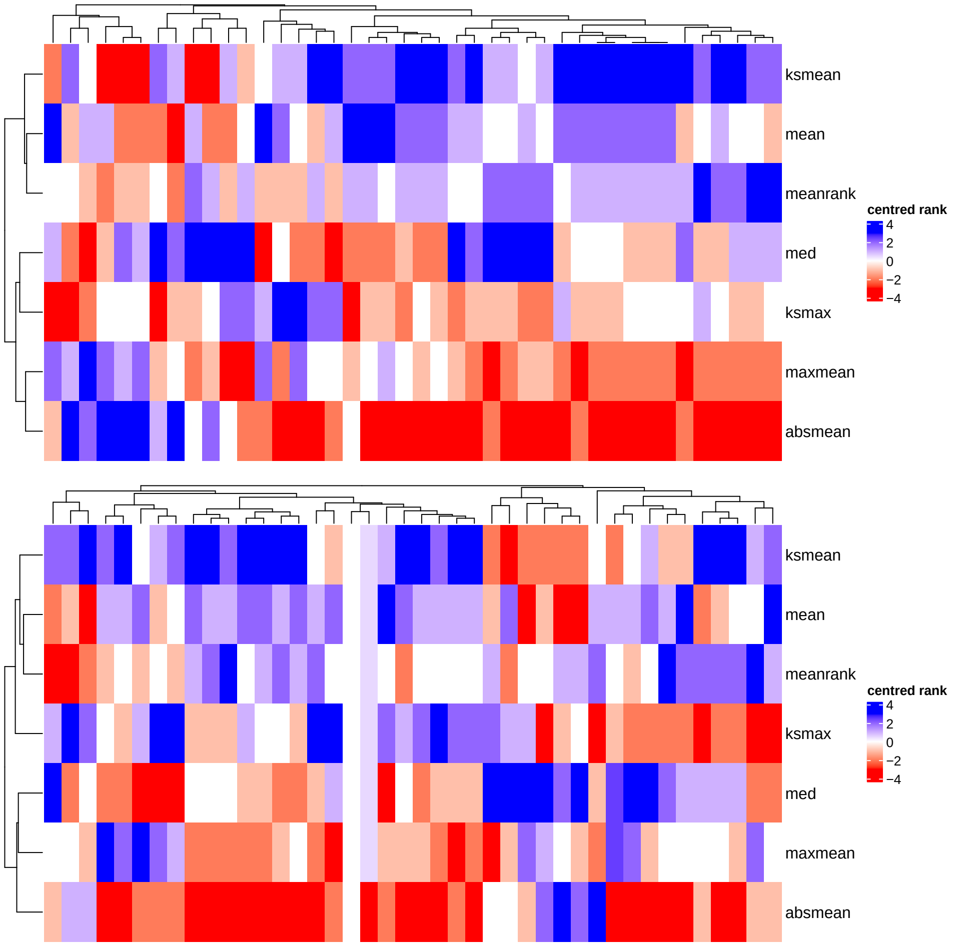

Figure S7: Heatmaps showing ranks (red -best rate- blue -worst rate-) in all 42 datasets from GEO2KEGG microarray compendium considering [top] measure M1 and [bottom] measure M2. The absmean finds the best scores in about half of the tested datasets. Even though the ksmean and the mean statistics present fairly poor results, they are able to achieve the best rates in a small group of datasets.

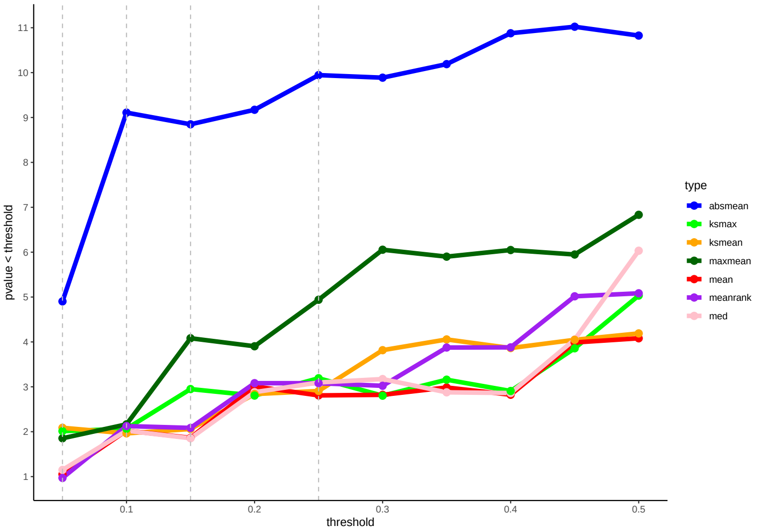

Figure S8: Number of datasets (out of the 16 RNA-seq-TCGA benchmarking datasets) with a M1 observed measure higher than expected by chance. In the x-axis there is the percentage of randomly generated rankings that present the same M1 score or higher than the observed score. The absmean finds the best results, with the other methods performing poorly.
