# Supplementary material for: Roastgsa: a comparison of rotation-based scores for gene set enrichment analysis
Source: BMC Bioinformatics. 2023 Oct 30;24:408. doi: 10.1186/s12859-023-05510-x (PMC10617084; doi:10.1186/s12859-023-05510-x)
Supplement: Supplementary file 2 — Additional file 2: supplementary table. [file 12859_2023_5510_MOESM2_ESM.pdf]

Scenario	Set of interest: $\forall g \in S$	Rest of genes $\forall g \notin S$
SC0	$\beta_g = 0$	$\beta_g = 0$
SC1	$\beta_g = \mathrm{N}(0.5, 0.1)$	$\beta_g = \mathrm{N}(0.2, 0.1)$
SC2	$\beta_g = \mathrm{N}(1, 0.1)\mathrm{I}(g \in C_1) + \mathrm{N}(0.2, 0.1)\mathrm{I}(g \notin C_1)$	$\beta_g = \mathrm{N}(0.2, 0.1)$
SC3	$\beta_g = \mathrm{N}(0.8, 0.1)\mathrm{I}(g \in C_1) + \mathrm{N}(-0.8, 0.1)\mathrm{I}(g \in C_2)+$ $\mathrm{N}(0, 0.1)\mathrm{I}(g \notin [C_1, C_2])$	$\beta_g = \mathrm{N}(0, 0.1)$
SC4	$\beta_g = 3\mathrm{I}(g \in C_o) + \mathrm{N}(0, 0.1)\mathrm{I}(g \notin C_o)$	$\beta_g = \mathrm{N}(0, 0.1)$
$C_1 \equiv$ subset of $\approx |S|/6$ genes from S<br>$C_2 \equiv$ subset of $\approx |S|/6$ genes from S, with $C_2 \cap C_1 = \emptyset$<br>$C_o \equiv$ subset of 3 genes from S		

Table S1: Specifications of simulation scenarios for microarrays data. The parameter β_g gives the effect size (or the log2FC), S denotes the testing gene set, and $[C_1, C_2, C_o]$ represent small subsets from the gene set S.

Scenario	Set of interest: $\forall g \in S$	Rest of genes $\forall g \notin S$
SC0	$\beta_g = 0$	$\beta_g = 0$
SC1-highcor	$\beta_g = \mathrm{N}(0.5, 0.01)$	$\beta_g = 0$
SC1-lowcor	$\beta_g = \mathrm{N}(0.15, 0.001)$	$\beta_g = 0$
SC2-highcor	$\beta_g = \mathrm{N}(2.5, 0.01)\mathrm{I}(g \in C_1) + 0\mathrm{I}(g \notin C_1)$	$\beta_g = 0$
SC2-lowcor	$\beta_g = \mathrm{N}(0.6, 0.01)\mathrm{I}(g \in C_1) + 0\mathrm{I}(g \notin C_1)$	$\beta_g = 0$
$C_1 \equiv$ subset of $\approx \|S\|/6$ genes from S		

Table S2: Specifications of simulation scenarios for RNA-seq data. The expected value and standard deviation specified here are included into the signal_params argument (thin_2group function from **seqgendiff** R package). S denotes the testing gene set, and C_1 represents a small subset from the gene set S.

n =		6	10	20	30	100
	sc.mean	45	53	50	47	55
	sc.maxmean	51	56	64	45	52
A1	sc.median	45	48	50	54	56
	sc.absmean	65	71	67	53	54
	co.mean	48	49	49	46	53
	co.maxmean	38	57	52	51	48
	co.median	45	45	56	47	49
	co.absmean	42	54	61	59	51
	meanrank	42	52	52	47	49
	ksmean	46	45	56	54	53
	ksmax	32	37	38	41	41
	sc.mean	57	55	61	65	53
	sc.maxmean	59	64	51	64	55
A2	sc.median	54	66	56	61	46
	sc.absmean	56	70	47	50	50
	co.mean	49	56	46	54	61
	co.maxmean	53	60	50	57	55
	co.median	52	67	49	55	48
	co.absmean	51	69	43	52	48
	meanrank	53	60	55	56	53
	ksmean	45	61	51	63	50
	ksmax	45	48	46	56	51
	sc.mean	70	61	45	51	41
	sc.maxmean	70	51	49	57	50
A3	sc.median	67	45	54	55	46
	sc.absmean	54	45	44	61	46
	co.mean	62	50	42	51	54
	co.maxmean	63	45	59	62	58
	co.median	74	54	55	53	59
	co.absmean	51	46	44	66	51
	meanrank	66	48	49	53	46
	ksmean	54	44	41	54	52
	ksmax	55	49	37	57	55

Table S3: Arrays simulated data: average empirical size (SC0) for all methods for $n = 6, 10, 20, 30, 100$ iid observations ($n/2$ per group) showing the number of times (from a total of 1,000 instances) that the test is rejected at a significance level of 0.05. The average intra-gene set correlation of A1, A2 and A3 are 0.05, 0.13 and 0 respectively. A value near 50 is expected for methods that control the false positive rate. This is fairly well controlled by all methods evaluated.

n =		6	10	20	30	100
	SC.mean	57	53	60	52	55
	SC.maxmean	53	46	45	53	55
	SC.median	61	49	48	60	55
	SC.absmean	46	39	48	61	43
A1	CO.mean	53	55	58	62	44
	CO.maxmean	50	58	52	65	51
	CO.median	50	59	60	52	51
	CO.absmean	47	38	54	59	45
	CO.mean.rank	52	61	53	54	42
	CO.ksmean	46	55	51	57	49
	CO.ksmax	50	54	60	61	51
	SC.mean	58	55	40	38	45
	SC.maxmean	58	49	40	37	43
	SC.median	63	54	42	39	45
	SC.absmean	50	50	45	40	49
A2	CO.mean	63	53	39	34	44
	CO.maxmean	62	57	39	39	44
	CO.median	64	50	39	35	34
	CO.absmean	60	55	38	31	41
	CO.mean.rank	68	56	41	34	43
	CO.ksmean	61	50	42	38	48
	CO.ksmax	59	49	48	42	43
	SC.mean	50	57	60	58	65
	SC.maxmean	52	49	55	55	57
	SC.median	59	48	58	59	68
	SC.absmean	56	49	54	49	60
A3	CO.mean	48	45	52	50	56
	CO.maxmean	63	49	46	43	53
	CO.median	50	47	60	43	68
	CO.absmean	46	59	48	41	62
	CO.mean.rank	47	50	53	50	57
	CO.ksmean	41	51	56	46	55
	CO.ksmax	47	43	49	43	52

Table S4: RNA-seq simulated data, average empirical size (SC0) for all methods for $n = 6, 10, 20, 30, 100$ iid observations ($n/2$ per group) showing the number of times (from a total of 1,000 instances) that the test is rejected at a significance level of 0.05. The average intra-gene set correlation of A1, A2 and A3 are 0.12, 0.25 and 0.35 respectively. A value near 50 is expected for methods that control the false positive rate. This is fairly well controlled by all methods evaluated.

n =		6	10	20	30	100
A1	mean	**156**	**338**	**578**	**776**	998
	maxmean	150	321	557	763	998
	median	143	287	509	715	995
	absmean	97	171	311	526	983
	mean.rank	157	322	574	774	997
	ksmean	167	318	571	770	997
	ksmax	74	213	483	713	997
A2	mean	**96**	**145**	**239**	334	**787**
	maxmean	89	144	229	322	767
	median	87	128	205	297	730
	absmean	71	125	191	264	703
	mean.rank	90	**145**	230	326	772
	ksmean	85	144	228	**337**	774
	ksmax	69	133	209	310	776
A3	mean	**348**	**640**	**926**	**988**	1000
	maxmean	311	587	904	977	1000
	median	210	410	787	911	1000
	absmean	117	171	435	610	998
	mean.rank	326	590	915	982	1000
	ksmean	297	567	899	979	1000
	ksmax	133	422	856	965	1000

Table S5: Proportion ($\times$ 1000) of tests that have been rejected with a rejection levels of 0.05. Scenario 1, arrays simulated data: same effect for all genes in the tested gene set, $n = 6, 10, 20, 30, 100$ iid observations (n/2 for each group). Only competitive test statistics are shown. The average intra-gene set correlation of A1, A2 and A3 are 0.05, 0.13 and 0 respectively. The mean statistic finds the best rates of all evaluated statistics, with the ksmean doing especially well in the A2 sets.

n =		6	10	20	30	100
	mean	91	137	210	296	651
	maxmean	111	159	278	392	762
	median	86	103	162	203	274
A1	absmean	**127**	**228**	**487**	**701**	**960**
	mean.rank	81	113	157	220	331
	ksmean	90	111	170	224	398
	ksmax	48	83	166	284	755
	mean	57	90	112	129	299
	maxmean	67	95	124	154	350
	median	63	73	83	99	128
A2	absmean	**86**	**143**	**198**	**303**	**811**
	mean.rank	50	70	80	93	105
	ksmean	54	72	86	96	117
	ksmax	51	68	95	116	310
	mean	241	374	635	756	905
	maxmean	**254**	**419**	**706**	**806**	931
	median	131	217	347	468	544
A3	absmean	182	338	643	791	**960**
	mean.rank	165	283	472	575	674
	ksmean	148	277	474	568	704
	ksmax	83	253	595	745	935

Table S6: Proportion ($\times$ 1000) of tests that have been rejected with a rejection levels of 0.05. Scenario 2, arrays simulated data: only a group of interconnected genes in the gene set have -a common- activity in the gene set, $n = 6, 10, 20, 30, 100$ iid observations (n/2 for each group). Only competitive test statistics are shown. The average intra-gene set correlation of A1, A2 and A3 are 0.05, 0.13 and 0 respectively. The absmean (in A1, A2) and maxmean (in A3) statistics find the best rates of all evaluated statistics.

n =		6	10	20	30	100
	mean	72	54	62	89	149
	maxmean	110	139	288	489	973
	median	60	53	71	98	123
A1	absmean	**270**	**488**	**834**	**962**	**1000**
	mean.rank	57	39	36	55	47
	ksmean	51	33	29	37	16
	ksmax	59	56	76	127	393
	mean	44	50	66	72	83
	maxmean	63	93	130	183	618
	median	41	51	66	63	75
A2	absmean	**111**	**255**	**513**	**737**	**993**
	mean.rank	27	40	35	26	14
	ksmean	30	35	29	23	5
	ksmax	39	49	59	68	67
	mean	117	150	282	387	613
	maxmean	269	495	877	950	998
	median	106	133	257	313	388
A3	absmean	**334**	**642**	**944**	**985**	**1000**
	mean.rank	78	95	148	203	267
	ksmean	58	65	82	106	117
	ksmax	131	189	411	562	948

Table S7: Proportion ($\times$ 1000) of tests that have been rejected with a rejection levels of 0.05. Scenario 3, arrays simulated data: two groups of genes, one up-regulated and the other down-regulated, are active in the gene set, $n = 6, 10, 20, 30, 100$ iid observations (n/2 for each group). Only competitive test statistics are shown. The average intra-gene set correlation of A1, A2 and A3 are 0.05, 0.13 and 0 respectively. The absmean statistic finds the best rates of all evaluated statistics, with the maxmean achieving the second best rates for all A1, A2 and A3 sets.

n =		6	10	20	30	100
	mean	57	69	64	70	97
	maxmean	71	78	96	133	187
	median	58	53	55	68	29
A1	absmean	**97**	**142**	**235**	**332**	**659**
	mean.rank	50	57	50	49	31
	ksmean	40	45	41	41	31
	ksmax	41	54	52	53	88
	mean	75	65	93	87	165
	maxmean	86	93	128	132	298
	median	65	58	43	51	37
A2	absmean	**135**	**189**	**339**	**431**	**939**
	mean.rank	60	49	43	45	31
	ksmean	40	37	33	32	22
	ksmax	47	55	68	57	115
	mean	460	711	942	979	1000
	maxmean	**710**	**930**	1000	1000	1000
	median	68	74	80	88	91
A3	absmean	640	928	1000	1000	1000
	mean.rank	75	90	115	112	123
	ksmean	82	107	112	115	135
	ksmax	507	805	982	998	1000

Table S8: Proportion ($\times$ 1000) of tests that have been rejected with a rejection levels of 0.05. Scenario 4, arrays simulated data: few genes -or outliers- present a much higher effect than the rest of the genes, $n = 6, 10, 20, 30, 100$ iid observations (n/2 for each group). Only competitive test statistics are shown. The average intra-gene set correlation of A1, A2 and A3 are 0.05, 0.13 and 0 respectively. The absmean (in A1, A2) and maxmean (in A3) statistics find the best rates of all evaluated statistics.

n =		6	10	20	30	100
SC1-highcor	mean	**234**	**436**	804	926	1000
	maxmean	232	420	800	**932**	1000
	median	200	376	754	888	1000
	absmean	220	410	766	906	1000
	mean.rank	236	410	**828**	914	1000
	ksmean	210	430	796	914	1000
	ksmax	192	400	784	922	1000
SC1-lowcor	mean	**150**	**276**	**588**	**750**	**988**
	maxmean	134	228	518	692	978
	median	100	210	430	588	946
	absmean	72	76	176	274	800
	mean.rank	122	262	536	730	988
	ksmean	132	242	520	714	982
	ksmax	114	226	488	708	980
SC2-highcor	mean	118	148	248	352	760
	maxmean	162	226	430	614	976
	median	58	56	56	58	116
	absmean	**360**	**562**	**900**	**948**	**1000**
	mean.rank	58	30	40	50	80
	ksmean	52	34	32	42	84
	ksmax	70	88	146	214	818
SC2-lowcor	mean	146	244	520	692	970
	maxmean	**162**	**356**	**688**	**868**	**1000**
	median	102	132	244	274	314
	absmean	120	234	552	780	**1000**
	mean.rank	118	184	328	458	566
	ksmean	116	180	300	418	584
	ksmax	156	280	532	748	990

Table S9: RNA-seq data, proportion ($\times$ 1000) of tests that have been rejected with a rejection levels of 0.05. Scenario 1: same effect for all genes in the tested gene set, $n = 6, 10, 20, 30, 100$ iid observations (n/2 for each group). Only competitive test statistics are shown. The average intra-gene set correlation of SC1-highcor, SC1-lowcor, SC2-highcor and SC21-lowcor are 0.22, 0, 0.28 and 0, respectively. The mean statistic finds the best rates for SC1 (with maxmean, meanrank and ksmean doing well in the SC1-highcor). The absmean (in highcor) and maxmean (in lowcor) out-power the other statistics in SC2.

	absmean	maxmean	mean	med	ksmean	ksmax	meanrank
M1	**2.45**	3.14	4.67	4.24	5.31	3.57	4.62
M2	**2.31**	3.64	4.49	4.02	5.08	4.06	4.39

Table S10: Average rank resulting from measures M1 and M2 using the 42 datasets from the benchmarking package. Rank 1 is the highest rate and rank 7 is the lowest rank. The absmean score achieves the best rates in the two measures followed by the maxmean approach. Only competitive scores are used.
