# Supplementary material for: Roastgsa: a comparison of rotation-based scores for gene set enrichment analysis
Source: BMC Bioinformatics. 2023 Oct 30;24:408. doi: 10.1186/s12859-023-05510-x (PMC10617084; doi:10.1186/s12859-023-05510-x)
Supplement: Supplementary file 3 — Additional file 3: some supplementary material: comprehensive description of rotation approach for geneset enrichment analysis; description of public data considered; comparison between absmean and mean statistics in simulated data; importance of gene coverage for roastgsa methods; and comparison between camera and roastgsa approaches in benchmarking data. [file 12859_2023_5510_MOESM3_ESM.pdf]

Additional file 3

The first two sections explain in detail the rotation approach for gene set enrichment analysis. These are written based on the results from work by Lansgrud (2004) [3] and Lim et al. (2010) [5].

1 Re-interpretation of linear models under QR decomposition

Let Y_i be a q-dimensional vector, independent for any $i \in [1, ..., n]$, that represents the gene expression profile of the ith sample with the following multivariate normal distribution assumption:

$$Y_i \sim \text{MVN}_q(X_i B, \Sigma_r),$$

where X is a $n \times p$ design matrix with $p - k$ adjusting covariates and k covariates of interest. Since the motivation of this paper falls in extending gene differential expression models at the level of gene sets, we look at the case where $k = 1$ such that the covariate of interest corresponds to the pth column of X. The $p \times q$ matrix B contains the linear regression coefficients and Σ_r is the error covariance matrix.

The matrix of covariates $X = X_Q X_R$ (by QR decomposition) can be decomposed by the product of the $n \times p$ orthogonal matrix X_Q and the $p \times p$ upper-triangular matrix X_R. Consider X_D and X_H the first $p - 1$ and pth columns of X_Q, respectively, with

$$Q = [X_Q \ X_E] = [X_D \ X_H \ X_E],$$

being the extended matrix of X_Q such that X_E is a $n \times (n - p)$ arbitrary matrix whose orthonormal columns are orthogonal to the columns of X_q. Note that

$$Y = XB + E = X_Q \tilde{B} + E, \text{ with } \tilde{B} = X_R B = \begin{bmatrix} \tilde{B}_D \\ \tilde{B}_H \end{bmatrix}.$$

Left-multiplying both sides of the equality by Q^t,

$$\begin{bmatrix} \tilde{Y}_D \\ \tilde{Y}_H \\ \tilde{Y}_E \end{bmatrix} = \begin{bmatrix} X_D^t Y \\ X_H^t Y \\ X_E^t Y \end{bmatrix} = \begin{bmatrix} I_{p \times p} \\ 0_{(n-p) \times p} \end{bmatrix} \begin{bmatrix} \tilde{B}_D \\ \tilde{B}_H \end{bmatrix} + \tilde{E}. \tag{1}$$

Here, the distributions of $\tilde{E} = Q^t E$ and E are identical with $\tilde{Y}_E$ containing $n-p$ independent observations of a $MVN_q(0, \Sigma_r)$. The q-vector of regression coefficients B_H is estimated by least squares with solution

$$\hat{B}_H = \tilde{Y}_H/(X_R)_{pp} = X_H^t Y/(X_R)_{pp}, \tag{2}$$

and associated standard errors

$$se(\hat{B}_H) = |(X_R)_{pp}|^{-1}(n-p)^{-1/2} \left(\sum_{i=1}^{n-p} (\tilde{Y}_E)_i^2 \right)^{1/2}, \tag{3}$$

on $n-p$ degrees of freedom.

2 Simulation of data under $\mathbf{H}_0$ using rotations

Obtaining test statistics under the null hypothesis, e.g., H_0: $B_p = 0$, can be done via a rotation on the residual space of the model in (1). As assumed above, the first $p-k$ columns of the design matrix X are adjusting covariates of no interest for testing, and so can be eliminated from the model. Rewriting the model formulation by

$$\tilde{Y} = \tilde{X}\tilde{B}_H + \tilde{E}, \tag{4}$$

where $\tilde{Y}$ and $\tilde{E}$ only contain $n-p+1$ rows, with the first row corresponding to the variable of interest, and $\tilde{X} = [1, 0_{n-p}]^t$.

Denote by R^* a simulated random rotation matrix, which can be obtained by taking the Q matrix of a QR decomposition of a $(n-p+1) \times (n-p+1)$ matrix which contains $(n-p+1)^2$ standard normally distributed random variables. As stated above, if

$$\tilde{Y}_i \sim \mathrm{MVN}_q(0, \Sigma_r), \quad \text{then } (R^*\tilde{Y})_i \sim \mathrm{MVN}_q(0, \Sigma_r), \tag{5}$$

follows immediately. If H_0 does not hold, the first row of $\tilde{Y}$ tends to have larger values which could lead to large magnitudes in the estimated coefficients under rotations and a decrease in the statistical power. This is

adjusted in the **roast** formulation by shrinking the values of $\tilde{Y}_1$ prior to obtaining test statistics upon rotations.

When the test statistic depends on $\tilde{X}$ and $\tilde{Y}$ trough $\tilde{X}^t\tilde{X}$, $\tilde{Y}^t\tilde{Y}$, $\tilde{X}^t\tilde{Y}$, finding the test statistics on new rotated data can be done via a rotation in the $\tilde{Y}$ matrix or a rotating in the $\tilde{X}$ matrix (even though $\tilde{X}$ is fixed by its nature). Rotating the $\tilde{X}$ matrix makes the approach computationally very efficient. As a fact, since $\tilde{X}$ contains only one element different from zero (the first), only the first row of R^* needs to be simulated. The newly estimated coefficients under a rotation of the matrix can be found by solving the next q least squares problem

$$\arg\min_{(\tilde{B}_H)_s} \sum_{i=1}^{n-p+1} (\tilde{Y}_{is} - R^*\tilde{X}_i(\tilde{B}_H)_s)^2, \ \ \forall s \in [1, q].$$

with solution

$$(\hat{\tilde{B}}_H)_s = \frac{\sum_{i=1}^{n-p+1} R_i^*\tilde{Y}_{is}}{\sum_{i=1}^{n-p+1}(R_i^*)^2} = \sum_{i=1}^{n-p+1} R_i^*\tilde{Y}_i, \tag{6}$$

or, equivalently, $\hat{\tilde{B}}_H = R^*\tilde{Y}$. The standard errors are also computationally fast to obtain:

$$se((\hat{\tilde{B}}_H)_s) = \frac{1}{n-p}\sum_{i=1}^{n-p+1}(\tilde{Y}_{is}^2 - R_i^*\sum_{j=1}^{n-p+1}R_j^*\tilde{Y}_{js})^2 = \frac{1}{n-p}\sum_{i=1}^{n-p+1}[\tilde{Y}_{is}^2 - (\hat{\tilde{B}}_H)_s^2]. \tag{7}$$

3 Public data sets

Metabric data. The metabric database contains the genomic profile and clinical information of 1980 patients with breast cancer [1]. We select the individuals with ER+ status.

GTEx data. Gene expression data with 286 samples from non-diseased mammary tissues are considered from GTEx (v1.1.9, SMTSD="Breast - Mammary Tissue") as part of our simulations strategy for obtaining RNA-seq data that could be similar to real case studies.

Gene set collections. The Hallmark gene set collection from MSigDB [4] is used to assess the performance of the gene set analysis methods presented

in this paper. We consider the interferon alpha response Hallmark as motivation for our proposed methodology since it shows the highest intra-gene set correlation of all 50 evaluated gene sets (Additional file 1:Figure S1).

Benchmarking data. A compendium of 42 microarray datasets investigating the KEGG specific functionality of human disease are compiled by [6] and made accessible through the R package **GSEABenchmarkeR** [2]. Moreover, TCGA data with the genomic profile of 16 cancer subtypes are also available through **GSEABenchmarkeR**.

4 Understanding the advantages and disadvantages of the absmean score

The absmean score obtained the best true positive rates of all tested scores, especially in the benchmarking data. Here we explore certain situations when this score fails to achieve good recovery rates in comparison to non-directional summary statistics such as the mean score.

In a simulations study, we pushed the absmean sensitivity to detect gene set effects with effect sizes varying from mild to strong and several percentages of activated genes. For the sake of simplicity, we assumed that for any gene g in the testing set S, independently, a differential expression value y_g can be obtained from a Normal distribution

$$y_g \sim a_g N(\mu, 1) + (1 - a_g)N(0, 1),$$

with a_g being a binary variable that can take values 0 or 1 with certain probability.

For this experiment, we fixed the signature size to $|S| = 50$ genes, varied $Pr(a_g = 1)$ from 0.1 to 1 to give different levels of active genes and varied μ from 0 to 2 to give different effect sizes magnitudes.

The hypothesis testing problem considered was the following:

$$H_0 : \text{avg}_S(E[y_g]) = 0; H_a : \text{avg}_S(E[y_g]) \neq 0$$

Null distributions were approximated by generating random sets of the same size as S under a standard normal distribution. For every combination of the pair of arguments $Pr(a_g = 1)$ and μ, we simulated 1,000 independent gene sets. The performance of the absmean and mean statistics was evaluated by comparing the proportion of gene sets (out of these 1,000 sets) with a test statistic larger than expected by chance ($\alpha = 0.05$).

We observed (Figure 1-3) that the absmean score lost power with respect to the mean score under the combination of both low effect size (within 1.5 standard deviations) and high percentage of activated genes (more than 30% of the genes).

5 Importance of gene coverage for roastgsa methods

Limitations with sequencing read depth in RNA-seq data impact the ability to characterize poorly expressed genes. Methods for gene-wise differential expression analysis such as DESeq2, by default, ignore genes that are lowly expressed in the multiple testing correction step. This is done because these lowly expressed genes are *a priori* unlikely to be called significant and would inevitably increase the adjusted p-value for the rest of the genes.

In the gene set enrichment step, true discovery rates improve when gene sets contain genes with high coverage (Figure 4). For this reason, genes with low coverage tend to be filtered out *a priori*, using thresholds that might be seen as arbitrary.

Here we explored the differential power of three **roastgsa** statistics (mean, absmean and maxmean) under SC1-lowcor and SC1-highcor models (Additional file 2:Figure 3) when several genes with low coverage are included in the testing signature. For that, we used a signature of 20 genes with a reasonably good coverage (basemean = 50 reads in all 20 genes) and we added into the signature 10 more genes with a specific coverage (going from 3 to 271 reads as basemean). We also kept the testing signature with the initial 20 genes as control. We considered $n = 10$ samples per group and a total of 1,000 instances per group.

The proportion of rejected tests (at 0.05 significance level) decreased when adding genes with a basemean of 3 reads, maintained similar powers with basemean of 5 and started to gain power consistently for larger basemeans, basemean ≥ 13 (Figures 5-6). For these data, a threshold of 5 could be considered a reasonable educated guess.

6 Comparing roastgsa and camera in benchmarking data

The **roastgsa** can be approached from a self-contained and a competitive scheme, with the latter forcing and hybrid of both gene centering and sample randomization. However, there are methods in the literature such as **limma**'s method **camera** that are presented as uniquely competitive by its definition. The **camera** approach can naturally be seen as a proper way to

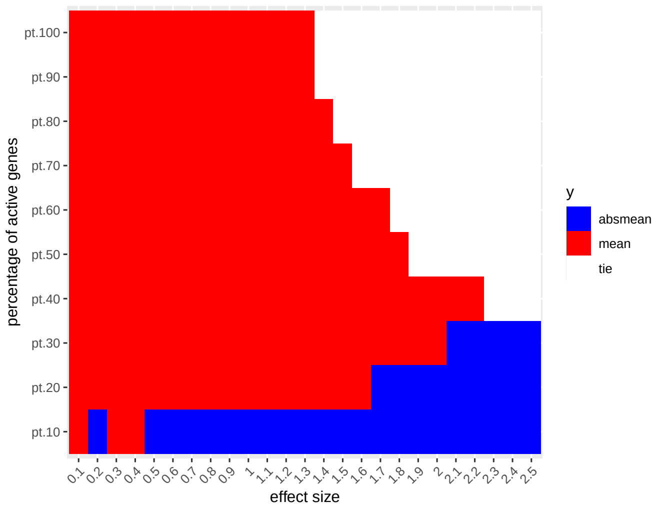

Figure 1: Evolution of power gain/loss of absmean score in comparison to the mean score for several percentages of activated genes and various effect sizes. In blue, the absmean score finds better rates than mean, and in red, the mean score finds better rates than absmean.

rank candidate gene sets in battery testing. Moreover, in such ranking-based interpretation setting, there have been certain concerns in the community about methods that impose a penalization related to the intra-gene set correlation levels of the tested set (and that is linked to the effective signature size concept discussed throughout the paper). On one hand, current versions of **camera**, by default, set a common (0.01) intra-gene set correlation in all gene sets, that favors highly correlated gene sets, with the aim to balance out an increase of false positives with a more comprehensible gene set ranking. On the other hand, in the **roastgsa**, which relies on the roast algorithm, the penalization for high intra-gene set correlated cases is maintained.

We examined whether the **roastgsa** scores produce comparable rankings to the **camera** approach using the benchmarking data. In figure 7 we show the percentage of times (out of the 42 datasets available) that **roastgsa** (with absmean, maxmean or mean measures) outperformed the **camera** approach. We find that the absmean and maxmean improved the rates of **camera** in more than 60% of the studies, with the mean presenting very similar ranking scores to **camera**.

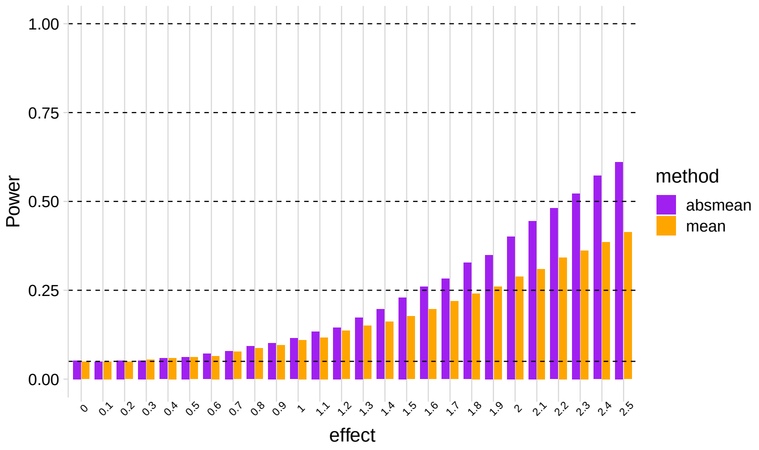

Figure 2: Gain of power (percentage of rejected tests at $\alpha = 0.05$) in absmean score in comparison to the mean score for 10% of activated genes.

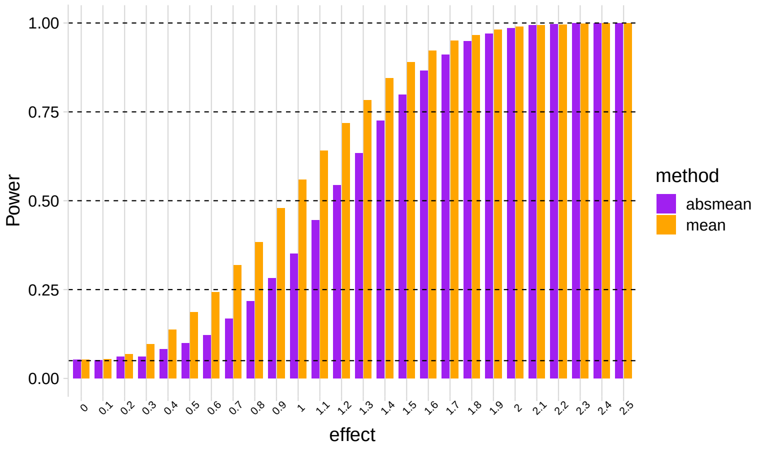

Figure 3: Loss of power (percentage of rejected tests at $\alpha = 0.05$) in absmean score in comparison to the mean score for 30% of activated genes.

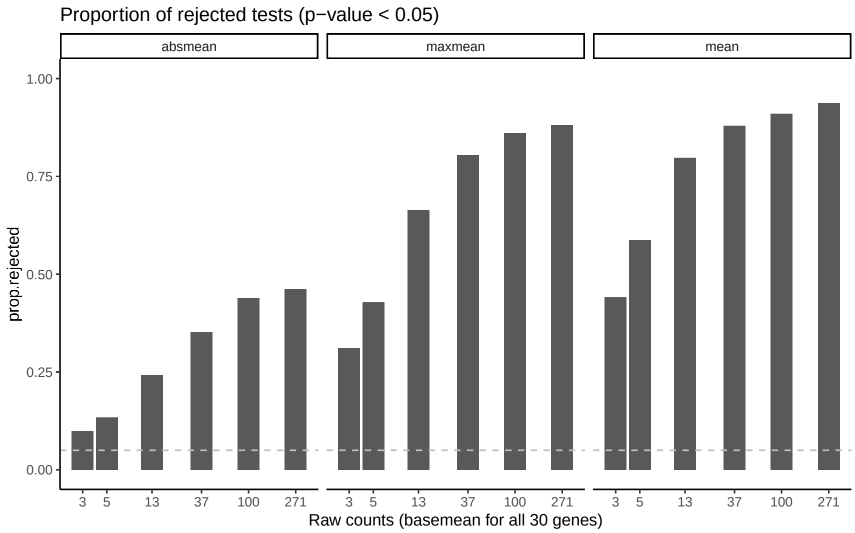

Figure 4: Differential expression model SC1-lowcor: Proportion of rejected tests ($\alpha = 0.05$) for gene sets of 30 genes with a certain coverage (groups of gene coverage are specified at x-axis). The proportion of correctly rejected tests increases with the read depth of the tested genes, for the three methods of GSA.

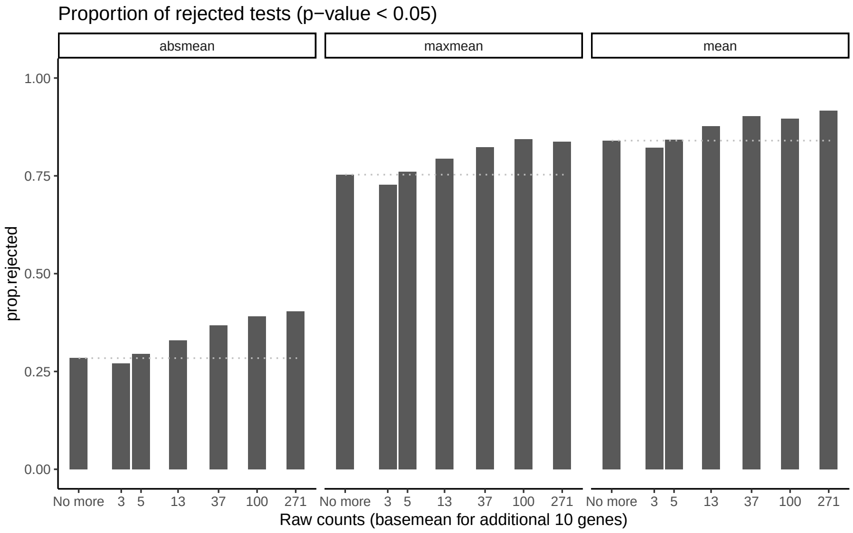

Figure 5: Differential expression model SC1-lowcor: Proportion of rejected tests ($\alpha = 0.05$) for gene sets of 20 genes with a basemean of 50 and additional 10 genes with a specific coverage. The coverage for the 10-genes subset is specified at x-axis. The proportion of correctly rejected tests increases with the read depth of the tested genes, with basemean of 3 reads reducing the power of the three methods of GSA.

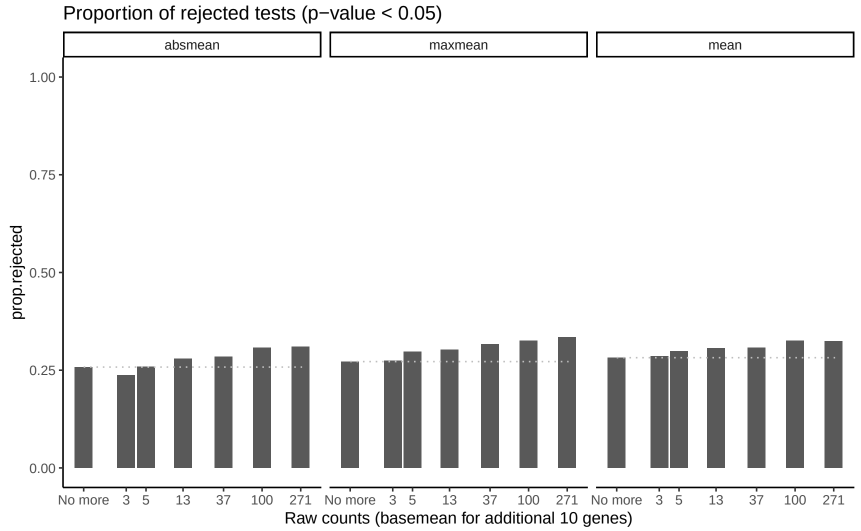

Figure 6: Differential expression model SC1-highcor: Proportion of rejected tests ($\alpha = 0.05$) for gene sets of 20 genes with a basemean of 50 and additional 10 genes with a specific coverage. The coverage for the 10-genes subset is specified at x-axis. The proportion of correctly rejected tests increases with the read depth of the tested genes, with basemean of 3 reads reducing the power of the three methods of GSA.

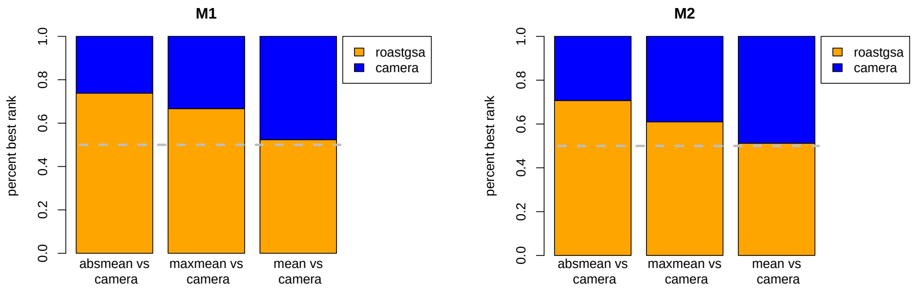

Figure 7: Percentage of studies (out of the 42 datasets) with higher M1 or M2 measure (left or right, respectively) in the **roastgsa** approach than in the **camera** approach.

References

[1] C. Curtis, S. P. Shah, S. F. Chin, G. Turashvili, O. M. Rueda, M. J. Dunning, D. Speed, A. G. Lynch, S. Samarajiwa, Y. Yuan, S. Gräf, G. Ha, G. Haffari, A. Bashashati, R. Russell, S. McKinney, S. Aparicio, J. D. Brenton, I. Ellis, D. Huntsman, S. Pinder, L. Murphy, H. Bardwell, Z. Ding, L. Jones, B. Liu, I. Papatheodorou, S. J. Sammut, G. Wishart, S. Chia, K. Gelmon, C. Speers, P. Watson, R. Blamey, A. Green, D. MacMillan, E. Rakha, C. Gillett, A. Grigoriadis, E. De Rinaldis, A. Tutt, M. Parisien, S. Troup, D. Chan, C. Fielding, A. T. Maia, S. McGuire, M. Osborne, S. M. Sayalero, I. Spiteri, J. Hadfield, L. Bell, K. Chow, N. Gale, M. Kovalik, Y. Ng, L. Prentice, S. Tavaré, F. Markowetz, A. Langerød, E. Provenzano, A. Purushotham, A. L. Børresen-Dale, and C. Caldas. The genomic and transcriptomic architecture of 2,000 breast tumours reveals novel subgroups. *Nature*, 486(7403):346–352, 2012.

[2] L. Geistlinger, G. Csaba, M. Santarelli, L. Schiffer, M. Ramos, R. Zimmer, and L. Waldron. *GSEABenchmarkeR: Reproducible GSEA Benchmarking*, 2019. R package version 1.2.1.

[3] Ø. Langsrud. Rotation tests. *Statistics and Computing*, 15(1):53–60, 2005.

[4] A. Liberzon, C. Birger, H. Thorvaldsdóttir, M. Ghandi, J. P. Mesirov, and P. Tamayo. The Molecular Signatures Database Hallmark Gene Set Collection. *Cell Systems*, 1(6):417–425, 2015.

[5] E. Lim, D. Wu, G. K. Smyth, M.-L. Asselin-Labat, F. Vaillant, and J. E. Visvader. ROAST: rotation gene set tests for complex microarray experiments. *Bioinformatics*, 26(17):2176–2182, 2010.

[6] A. L. Tarca, G. Bhatti, and R. Romero. A Comparison of Gene Set Analysis Methods in Terms of Sensitivity, Prioritization and Specificity. *PLoS ONE*, 8(11):e79217, 2013.
